# Women Face to Fear and Safety Devices During the COVID-19 Pandemic in Italy: Impact of Physical Distancing on Individual Responsibility, Intimate, and Social Relationship

**DOI:** 10.3389/fpubh.2021.622155

**Published:** 2021-03-12

**Authors:** Rosa Parisi, Francesca Lagomarsino, Nadia Rania, Ilaria Coppola

**Affiliations:** ^1^Department of Economics, Management and Territory (DEMeT), University of Foggia, Foggia, Italy; ^2^Department of Education Sciences, School of Social Sciences, University of Genoa, Genoa, Italy

**Keywords:** COVID-19, physical distancing, social responsibility, health citizenship, Italy

## Abstract

The COVID-19 pandemic of 2020 in Italy had its first epidemic manifestations on January 31, 2020. The socio-sanitary rules imposed by the government concerned the social distance and management of intimate relationships, the sense of individual responsibility toward public health. Physical distancing and housing isolation have produced new representations of intrafamily, generational, neighborhood, community responsibility, bringing out a new “medicalized dimension” of society. In light of this contextual framework, the research aims are to analyze how: the perception of individual responsibility for public and familial health and physical distancing has redrawn the relation between subjects-family-community; the State's technical-health intervention has reformulated the idea of social closeness, but also how the pandemic fear and social confinement has re-evaluated a desire for community, neighborhood, proximity; during the lockdown families, friends, neighbors have reconstructed feelings of closeness and forms of belonging. The methodology used is quanti-qualitative and involved 300 women through an online questionnaire. The data collected highlight how the house during the lockdown is perceived as a safe place and how women implement both the recommendations and the behaviors aimed at preventing contagion, but also ways that allow coping with the situation from a perspective of well-being. Furthermore, the data show how the dimension of distancing has loosened the relational dimension outside the family unit, with a greater distancing compared to pre-pandemic data. However, the majority of women report that they have joined solidarity initiatives, demonstrating that they want to maintain ties and participate actively in community life.

## Introduction

The world emergency that emerged with the COVID-19 contagion has brought out numerous reflections on these aspects, in particular with respect to the relationship between security, care for the weakest and intergenerational relations that are realized starting from the idea of a sort of “health citizenship” (1) where access to resources is granted to those who fall into behavioral patterns of protection against risk. Despite the differences in the different countries, in many cases the institutional health policies have rewritten the intergenerational pact promoting care through physical distancing. Distancing care is a well-known issue in transnational families (2–4), where literature has long reflected on the implications relating to the difficulty of reconciling care and physical distance; a theme that up to now has concerned migrants, while it was totally unknown to families living close by. Among the most effective slogans of the social persuasion campaign to adopt socio-sanitary norms were: “Distant but united.” Physical distancing and housing isolation have produced new representations of intrafamily, generational, neighborhood, community responsibility, bringing out a new “mediatised dimension” of society.

The pandemic emergency has radicalized the trends already in place in society and produced new and unexpected reconstructions of the social bond, and of the public dimension of individual responsibility. In the pre-COVID society, studies had highlighted the growth of the individualization process, both in the family (5–7), and in society (5, 8, 9).

Social and health policies to combat COVID-19 on the one hand imposed the need for physical distancing and isolation, while on the other, it brought to light the “removal” of the community, of solidarity, from family and relatives to that of the neighborhood, and even on a national level. The confinement has caused many psychological strain that has leaded, for example, to alters physical activity and eating behaviors in a health compromising (10). In many cases, these effects have been mitigated through the use of technology that has allowed the opportunity for social relations to be maintained (11). During COVID-19, there have been many episodes of reconstruction of micro neighborhood relationships (support for the elderly in shopping, exchange of conversations between neighbors from balconies, community singing), of building familiar and friendly communities through web devices.

The fall of the myth of the omnipotence of biomedicine has paved the way toward the perspective of considering medicine as a collective phenomenon and therefore of common interest (12, 13). The knowledge of biomedicine centered on technology and technique has been overwhelmed by a shock of reality and has given way to simple rules of common sense: wear a mask, clean and disinfect your hands frequently, avoid close contact, keep a physical distance of at least 1 m, sneeze or cough into the crook of your elbow. Public health policies to combat the pandemic have wagered on people's adherence to the rules of containment and social distancing: a meter has become the measure of our sociality. Clear rules of common sense which, as Beneduce (14) observes, derive from a “common-trivial, intuitive or feminine knowledge.” The reference to the world of women with respect to self-care and others, takes up the focus of our article aimed at investigating the role of women and women's actions in response to health as a “collective good of common interest.” Indeed, research carried out on eight countries, including Italy, showing how woman are more careful to spread and take steps in adopting behavior imposed by the state to protect herself and others, and so to adopt more altruistic approaches (15). According to Cheng, Lam and Leung (16) awareness of governments and the WHO on the massive use of masks has shifted attention from protecting oneself to protecting others, taking the form of altruism and solidarity.

The measures adopted, such as containment, distancing, and personal protection, in the early period of the pandemic were not accompanied by policies of tests and targeted isolation or tracing the contagion, meaning that people were confronted with the burden of responsibility for the success of public health policies. A situation well-condensed in the expression “we are healthcare,” circulated during the most dramatic moments of the pandemic. This responsibility is configured in its double dimension, ethical-moral and juridical. In fact, scrupulously adopting the provisions indicated by the Government becomes indicative of being a virtuous citizen, worthy of health citizenship. Following the rules testifies to fidelity to the “collective pact” to save public health (17), but at the same time it is also a duty, because non-observance of the rules is punished with sanctions. Therefore, adhering to the “collective pact” means acting responsibly for the protection of collective health. The speech by Italian Premier Giuseppe Conte in the press conference in which he announces the lock-down is a sort of founding act of the “collective pact,” the only tool to deal with a “new” virus of which “there is no great scientific evidence” and therefore there are no known medical cures for healing. In the words of the Premier, adherence to the “collective agreement” calls for a direct, emotional, sentimental involvement of people called to safeguard public health through responsible action aimed first of all at their most fragile loved ones (parents, grandparents). In this message, the idea appears that citizens exposed to risk must be protected by the same community, organized in concentric circles of proximity, where the one closest to the subject coincides with the group of loved ones (partners, children, family members) then that of friends, neighbors and gradually on toward the national grouping. Furthermore, the feeling of participation in a larger, national community is strengthened as it drags with it the sense of fidelity to a pact built in the sphere of the most intimate affections. Clearly, this is an “unexpected” idea of community, musicalized through a social new order based on “staying at home” and on social distancing.

## Variable Geometry Quarantine: Public Health Politics and Regulatory Device in Italy

On 29 January 2020, a couple of Chinese tourists were rescued from a hotel in Rome by an ambulance with nurses dressed strangely in protective suits and white overalls. The whole of Italy was dismayed by such unusual and apparently out of place images. On 30th January, the Italian government proclaimed a state of emergency with consequent measures aimed at containing the infection throughout the national territory. Attitudes, body postures, lifestyles that have always been considered natural enter a shadow. The contagion of the virus feeds on social proximity. Kissing, hugging, greeting each other with a handshake, relaxing with friends, having a dynamic life are stigmatized as behavior in conflict with the protection of public health. Italy thus entered a path of progressive regulatory restriction of everyday behaviors that up until then had regulated social life, even in the most intimate aspects of the manifestation of affectivity and sociality. The Decree of the President of the Council of Ministers (DPCM) shows that medicine and political power “continue to intersect” and exercise a power that penetrates “invisibly into bodies, into behavior, shaping our experience” (14, 18). On the other hand, local adaptations, generally in a more restrictive sense than government measures aimed at social containment and distancing, show the centrality of “principle of responsibility” as a “principle of political action” (19).

We are interested to emphasize the function of the DPCM and institutional communication to support the rules against COVID-19, in identifying scales and methods of responsible action. The house, the perimeter around the house, the neighborhood, the places of basic necessity (supermarkets, pharmacies), the country in which you live constitute the boundaries of a progressive cartography within which to circumscribe social action at the time of the COVID-19. Sociality protected by masks and physical distancing constitutes the form within which to continue a safe social life for oneself and for others. The containment and distancing measures have a progressive trend that heads down from the international context and toward the domestic. Within a few weeks, the first outbreaks broke out in two regions, Lombardy and Veneto. The DPCMs, which then followed on constantly until early March, progressively extend the “red zones” from the North to the rest of Italy and follow the degree of alert of the pandemic globally. On 4th March, schools and universities closed all over Italy. Until 11th March when, in conjunction with the WHO declaration of a state of “global pandemic,” Premier Giuseppe Conte announced with a live TV broadcast and Facebook post that he had signed the DPCM whereby the “red zone” was extended to include the whole of national territory. Italy came to a standstill. The home and the co-residence family community represents a safe place from various points of view from aspects of sociality and psycho-physical well-being, economic, emotional and affective one to the hygienic and food safety point of view (20). So, family becomes a safe place capable of controlling and preventing contagion. However, according to literature, family relationship inside home can be a protective factor or a condition of fragility within which the health of its members develops (21, 22). Moreover, because the family is system characterized by interdependent relationships (22, 23) the well-being or the malaise of one of its members affects other individuals. The family environment has a relevant not only for psychological health and quality of life, but also for individual adaptation and well-being of different members (24, 25). In actual fact, the forced coexistence during the lock-down period has also highlighted extreme situations of conflict, sometimes resulting in violence against women or children (26, 27). Furthermore, the communication campaigns that preceded and reinforced the regulatory provisions of the lock-down were based on direct and to-the-point slogans, among the most used: “I'm staying at home,” that refers to a sense of responsibility and self-discipline. Progressively, the “boundaries of everyone's world have narrowed more and more, until they coincide with the walls of our homes” [(28), p. 76].

## From Family Community to Health Citizenship

In contemporary society, a feeling of nostalgia prevails, which accompanies the loss of the community of the past, idealized as place where relationships were immediate and supportive (29–31). Clearly, this is an idealization, since the communities of the past had many contradictions and paradoxes, the first being that of the relationship between safety and freedom (30). This contradiction is among the first factors to ensure that the communities of the past cannot return. Nonetheless, the idea of community in recent years has made its way unexpectedly, so much so that it is not immediately recognized. One of the characteristics of contemporary communities is that they are not tied to a territory, they are nourished not so much by face-to-face relationships but by “virtual neighborhoods” or transnational landscapes (32). The Social Street, for example, is a group of fellow citizens who meet for the achievement of a common advantage, it was born on the web, uniting people who do not know each other but who live on the same street. A recent research highlights how women, which belong to the Social Street, can become promoters of psychological well-being and healthy communities (33). Other examples are patrimonial communities, among all patrimonial food communities, or the communities constitutes for the protection of “common goods.” How this trend has been further increased, however modified, under the push of medicalization of the social, of physical distancing, of the sense of fear toward the closest people considered as potential carriers of contagion.

Palumbo (34) considers the pandemic a “hybrid” and its effects include that “of staging a return of the social,” in the “re or hyper-mediatized” form. The re-emergence of the social crosses the theme of the community in the sign of hybrid and paradox. Migliorati (28) focuses on two types of communities that advance: one that, on the proposal of singing, playing, speaking from balconies, refers to the recovery of an “old ancient world” [(28), p. 73], destined for the most part to fail as prophesied by Bauman (30). In fact, these proposals have mainly had a media life on social networks and have mostly constituted an attempt to represent a national community made up of neighborhoods that adhered to the government slogan “everything will be fine.” Migliorati (28) reports a funny voice message on WhatsApp circulated during the lock-down period that says: “I have been locked up at home with my family since yesterday; they seem like good people” [(28), p. 72]. Clearly, this is a joke, which shows how often, in fact, our hectic lives do not allow us to devote sufficient time to cultivating family relationships. Therefore, if, on the one hand, the nostalgic recovery of forms of sociality based on a community model of the past is destined to fail, the imposition of staying at home has forced everyone to stop and devote more time to the family (28). But, as Smith et al. (35) emphasize, COVID-19 highlights the Connectivity Paradox of staying connected but distancing which leads “distanced connectivity.” Family communities are thus segmented internally, separating those who are in good health from those who are medically fragile. This division separates the generations and invites the younger to be responsible toward the older ones. A responsibility that paradoxically manifests itself through physical but not emotional distancing. The distancing from loved ones is presented as a necessary sacrifice, an act of love. One of the slogans of the institutional communication campaign is “keeping grandparents away to embrace them later.”

A final interesting aspect concerns the way in which health policies cross the theme of the community in the dimension of health citizenship. It presents itself as a form of belonging that redesigns the relationship between individuals and the State and has changed the sphere of personal rights. Health citizenship is increasingly present in the public and scientific debate in relation to disease prevention and health management in the context of profound demographic, ecological, economic, and political change (36). The relationship between health citizenship, rights and responsibilities changes over time and determines different configurations of public health systems and practices. In particular, the social meaning of health citizenship changes in relation to the role of public health in the construction of states, the theories on the healthy body and the role of biological determinism in the construction of subjective identity and the rights to health (36). Citizens who actively participate in the “collective pact” for the rescue of public health enter the sphere of health citizenship, as we have already specified in the previous paragraph. Otherwise, those who do not adhere to the new public security order are marginalized or stigmatized (37). One example is the public debate of stigmatizing the behavior of university students from the southern regions who study in northern Italy. On 8th March, Premier Conte announced the ban on moving the areas of northern Italy included in the red zone. A few hours before the official publication of the DPCM, the news spreads on social media. Many students and workers originally from southern Italy residing in the north, “attacked” the trains to return to southern Italy and their families. The public debate, the newspapers stigmatized those who returned from the north seen as a “smearers” and models of “bad” citizens. The south region governor of Apulia declared: “you are bringing us many other outbreaks of contagion that we could have avoided. (…) I remind you (…) that you must stay away from parents, siblings, grandchildren, friends, grandparents and sick people who risk dying if infected.” Indeed, responsible action is constituted as an act of citizenship (38) that expands the rights of health citizenship beyond the established limits toward levels of greater inclusion.

## A Gender Perspective: Women and the COVID-19 Pandemic

In literature, when we relate to gender, we refer to roles, responsibilities, and power relationships that are socially constructed and assigned to men and women in a given society or community. Gender perceptions are deeply rooted, vary widely within and between cultures and change over time, however in all cultures, gender determines power and resources for women and men (39–42). As Bond et al. (43) state, the focus on gender role development can be considered one of the most important areas of community development. In agreement with Rollero et al. (44) paying attention to gender equity affects many indicators of community life including the well-being of the community itself, making it more competent and capable of creating human and social capital. The attention to the development and well-being of the community in its sociological and psychological components is based on a situated analysis of human behavior and is particularly suitable for the analysis of gender as a context, in an inter-sectional perspective, in which the spaces of intersection of power relations are evaluated (45). In addition, focusing on the community gender dimension makes it possible to understand what is happening and to promote and produce social change. Just as Lewin's teaching on changing eating habits started from the guardians of food, so too, in this pandemic situation, understanding how women act and what they do to protect health can become an element for implementing social changes that “do not arise within an empty space but are part of the daily rhythm that pulsates between alternating sleep and wakefulness; of solitary and group life; of play and productive work; of belonging to a city, a family, a social class, a religious group, or a nation” [(46), p. 30]. According to Levine and Perkins (47), every sustainable social organization has structures and meanings that ensure its continuity in spite of environmental vicissitudes. Although much research shows that women suffer greater malaise and are more fragile in relation to this pandemic situation (48, 49) however, there is a lack of work from a gender perspective showing the condition of women in relation to the experience of distance and how this condition has brought about changes in family and community relations and has seen women themselves as active protagonists of solidarity actions and individual vs. collective responsibility. In order to ask for more attention and reflection and promote policy interventions for health and to consider the contribution of women to the health of the community (50). Moreover, the importance of attention to the involvement of people in their own health choices is now known (51, 52) and how the changes introduced by new health technologies make the relationship with health more predictable, which in this pandemic situation, on the other hand, seems to waver but, at the same time, require the person to internalize the idea of inevitable risk (53, 54), which in this context becomes even more pervasive.

## Aims

Based on this theoretical framework, and pandemic socio-political condition, we intend to investigate in a gender intersectional perspective the impact of physical distancing, within family and community relationships during the end of first quarantine period that the Italian population found itself facing (April–June). The focus on the role of women in respecting and promoting the rules of containment and social distancing, and how the perception of female individual responsibility for public and familial health and physical distancing redraws the relation between subjects-family-community and how the vision of health as a collective good to be protected extends health citizenship and has changed the sphere of personal rights. Moreover, in light of these changes, considering to family relationship and family conflict are other significant dimensions to investigate.

## Participants

The sample numbered 300 Italian women, distributed throughout the national territory, with an average age of 41.40 years (SD = 15.51, range 18–83). Regarding marital status, half of women (50.3%) declare that they are married/cohabiting, while 39.6% are single, 8.7 % are separated/divorced and 1.3% are widows. Considering the people with whom women live during the COVID-19 emergency, 14.1% say they are alone, while 23.9% live with another person, 30.6% with two other people, 23.6% with three other people while 7.7% live with more than four people. Moreover, women living with one or more people, in 63.3% of cases live with their families, in just 22% with a partner and 4.7% with friends or housemates.

Most of the women have a university degree (41.1%) or post-graduate qualification (19.7%), while 36.1% have a secondary school diploma and 3.1% have finished middle school. Regarding the family income the participant declares in 16% of cases up to € 15,000, in 37.1% of cases between 15,001 and 28,000, in 32% between 28,001 and 55,000, in 10.2% of cases between 55,001 and 75,000, while only the 4.8% declare a family income of over 75,000.Most of the participants (55.6%) live in a large city (more than 100,000 inhabitants), while 24.2% live in a medium-sized town (between 10,000 and 100,000 inhabitants), while 20.2% live in a small town (fewer than 10,000 inhabitants).

In respect of income during COVID-19, 65.8% of women declare they have an income equal to before, while only 1.3% declare they earn more, instead 32.8% declare an income lower than before and in half of the cases sustained by state aid. The majority of women switched to smart-working (59.8%), with only 14.1% continued to carry out the previous activity in the same way, while 13.6% said they had undergone a reduction or a change of hours while the 11% say they have asked for COVID or parental leave or been laid off; only 1.5% have undergone a change of role.

## Method and Measure

The method used is a quantitative approach, the questionnaire of an exploratory nature, it follows recent reflections on the design and application of online questionnaire surveys (55). It included some areas that were identified after holding focus group meetings. The questions are based also the results of earlier research that the authors were developed during the first period of lock-down due to the pandemic COVID-19 that have involved 1,250 participants (49).

The dimensions further analyzed are:

Behavior to protect health and social distancing

- The questions related to this area were intended to investigate how women behaved to protect their health and what kind of social distancing they adopted with the people who lived with them. Both questions were multiple choice with multiple answer alternatives to choose from.
- The “Inclusion of the Other in the Self” (IOS) (56, 57): the scale is a simple pictorial tool, which is consist in two increasingly overlapping circles indicating the degree of proximity to each other. One circle represents your own self, and the other circle represents the self of another individual. We have chosen this scale to evaluate the level of closeness/social distancing between the respondent and another individual. The IOS task asked respondents (“You” in our version) to assess their relationship with a specific individual (referred to as “X” in figure proposed) by selecting one out of seven pairs of increasingly overlapping circles. In each pair of circles, one circle refers to the respondent and the other circle to X. Respondents were asked to select the pair of circles that best describes their relationship with X. For example, if a respondent feels unrelated to X, it would be natural to select the pair of still separate circles; if a respondent feels very close to X, he or she may choose the almost completely overlapping set of circles. In our study the scale was used in four versions in which the X represented: a person very close to the compiler, a neighbor, a good friend, a resident of the same neighborhood where the subject lived. The respondent had to indicate the image that most represented the term “we” to define himself and a person that she/he considered very close to himself during the lock-down by selecting the pair of separate circles from 1 (not very close) to 7 (very close), that is, the overlapping circles.

Maintaining family relationships

- The questions relating to this area were intended to investigate family relationships during the period under investigation, with attention paid to the sharing of spaces (multiple choice question with only one alternative answer to choose from and an open question in which the choice given) and moments of conviviality and socio-relational (multiple choice question with multiple alternative answers to choose from).

Neighborhood relations and collective solidarity initiatives

- This area intended to investigate neighborhood relations during the lock-down and participation in collective and/or solidarity initiatives. The two questions were formulated with different answer alternatives among which the respondent could choose more than one.

Family conflict

- Presence or not of conflict in the family with dichotomous question (yes/no).
- Causes of conflict within the family during the lock-down: multiple choice question.
- How the conflict arose during the lock-down: single choice in multiple choice question.
- Thinking about the period they lived before the pandemic, respondents had to indicate on a scale from 1 (not at all) to 5 (very much) the level of conflict between them and the people with whom they lived (partners, children, parents, siblings).
- Thinking about the period experienced during the lock-down, respondents had to indicate on a scale from 1 (not at all) to 5 (very much) the level of conflict between them and the people with whom they lived (partners, children, parents, brothers/sisters).

Socio-demographic variables

- Socio-demographic questions: age, gender, civil status, educational qualification, age range of children, type of work during the COVID-19 health emergency, income.

## Procedure

The questionnaire was proposed on-line, the research team sent a link by e-mail, WhatsApp, discussion forums and social networks such as Facebook to reach a larger number of women. The inclusion criteria were being at least 18 years old and living in Italy during the lock-down due to COVID-19. The sampling was random cascade and it started with women known to the researchers; hence, the sample is of convenience.

The ethics committee of the Department of Education Sciences of the University of Genoa approved the questionnaire, and the data was collected in compliance with privacy rules and the research ethics code of the Italian Association of Psychology. During the last week of the lock-down the researchers collected the data, after people had stayed 40 days in isolation in their homes. The 1st day on which the questionnaire was disseminated, it was completed more than half by participants. This data is in line with other research conducted on-line during the COVID-19 pandemic (58, 59). People took ~22 min to fill it out. At the start of compilation, there was information regarding the research objectives, the areas investigated, the type of return, informed consent, and the method for withdrawing from the study.

## Data Analysis

Descriptive statistics were calculated for sociodemographic characteristics and information about variables, while the IOS scale scores were expressed as means and standard deviations. Moreover, to compare the differences between the results to IOS scale of our participants in relation to the pre-pandemic data (57) *t*-tests was conducted for single samples. The verification of the normal distribution of the sample was first done. *T*-test for paired samples, on the other hand, was used to analyse the difference in means in relation to the variables: family conflict (before and during lock-down). The Cohen's *d* was used to calculate the size of the effect. Finally, Chi-square analysis was performed to investigate the relation between causes of conflict and activities carried out with children and children's ages. All tests were two-tailed, with a significance level of *p* < 0.05 or *p* < 0.01. Statistical analysis was performed using SPSS Statistic 18.0. The qualitative open question was analyzed by two independent judges following the constant comparison analysis technique (60). The approach is based on grounded theory (61) and is supported by the use of the software Nvivo12 (2018).

## Results

### Women and Health Protection During COVID-19: Behavior and Social Distancing

When asked “how do you behave to protect your health” women, in most cases (89.3%), use personal protective equipment when they go out and limit outings (75.7%), followed by: they wash their hands often (65.0%), do physical activity at home (45.7%), frequently sterilize environments and objects (32.3%), do not touch eyes, mouth, nose, ears (20.3%), check the behavior of those who live with them (20.0%), spend a lot of time in isolation in their room (6.3%), never go out (5.7%), buy only packaged things (3.7%), use personal protective equipment even when they are at home (1.7%). Considering only women living with other people in the house (*N* = 260), it emerges that in most cases (76.9%) women do not apply any kind of distancing with their family members, while 16.5% avoid kisses, 14.2% do not embrace those who live with them, 4.2% avoid sex with their partner, 2.3% live in separate rooms from others, 1.9% keep 1 m away at home, 0.4 use a mask at home.

Analyzing the data in relation to the IOS scale it emerges that the sense of closeness and the sense of “us” are higher when people refer to a close person (M = 5.0; SD = 2.0), followed by a good friend but with an average lower than the theoretical average (M = 2.74; SD = 1.94), while the neighbor and a resident of the neighborhood obtain, respectively, lower scores (M = 1.75; SD = 1.35; M = 1.47; SD = 1.06). Comparing our data with regulatory data (60) it emerges that there are no significant differences with the person considered close (M = 5.2, SD = 1.3) while both the neighbor and the resident of the neighborhood obtain lower proximity scores compared to the data relating to the Gächter study (57) with statistically significant differences in the *t*-test per single sample [neighbor *t*(298) = −7.04, *p* < 0.001 Cohen's *d* 0.42; inhabitant of the neighborhood *t*(298) = −13.5, *p* < 0.001, Cohen's *d* 0.70]. In both cases we compared our data with the figure of the acquaintance of Gacher's study (M = 2.3, SD = 1.3). Also, with regard to the figure of the friend during the lock-down period, the score obtained on the IOS scale is significantly lower than the measurements taken in non-lock-down periods [M = 2.74; SD = 1.94 vs. M = 3.70; SD = 1.30; *t*(298) = −8.61, *p* < 0.001; Cohen's *d* 0.58].

### Maintaining Family Relationships

As regards family relationships, it emerges that in most cases women declared that it was better to live with their family members (52.8%) or in a couple with their partner (27.8%), and only a small part only with children (3.7%), with friends (7.4%), or alone (8.4%). If we analyse the qualitative reasons behind the choices made by women, the idea emerges that, at a time like that of the lock-down, family relationships make it possible to overcome loneliness, keep company and take care of each other. In fact, among the prevailing motivations of those who answered “better to live with family” there are elements that refer to the idea of the family as an emotional place, of sociability, mutual care, psychophysical well-being, emotional stability, contrasting stress, loneliness, and the onset of depression. The family also generates trust in the other, in their adherence to the virus protection and containment rules, representing, for the respondents, the best and most functional group of mutual protection from contagion.

This perception transpires both from those who live only with the partner and from those who live with the partner and children but also for adult children who have been with their elderly parents. As also emerges from the research carried out during the same period by the University Center for Studies and Research on the Family, Catholic University of the Sacred Heart [(62), p. 36]: interpersonal family relationships are configured as a reservoir of sociability and trust [.] in short, families even in a period marked by objective criticalities, they are able to grasp the positive added value of the bonds in terms of share capital.

It is also interesting to note that a certain awareness emerges about the difficulties of relationships in this moment due to the forced coexistence, with some women very clearly underlining the gap between the ideal perception and the real difficulties of coexistence, highlighting the risk of tensions and conflicts where relationships were difficult before. Mirroring this, those who answered “better to live alone” (8.4%) specified, in fact, that the choice is linked, in addition to avoiding the stress of often conflicting cohabitations or in confined spaces, to the fear of the risk of being infected or of infecting “In the family one is less alone but more risky, better alone”; “Less chance of spreading the virus to elderly or at-risk relatives,” “We avoid accidentally infecting sections of the population at risk.”

The women highlighted how the moments of greatest sharing in the family were linked to: eating meals (89.8%), watching TV programmes (70.5%), cooking together (63.1%), playing board games (46.1%), sports (26.4%), gardening (23.7%), musical activities (singing, dancing) (20.0%), seeking information on COVID-19 (18.6%), praying together (8.8%) and meditation (3.3%).

### Family Conflict: Reasons, Ways, With Whom

In relation to the dimension of family conflict, 73.4% declare that they have experienced this, in particular a relationship emerges between the conflict and the age of the children of the women [χ^2^(5) = 15.81, *p* = 0.006, Cramer's V = 0.36]. The highest percentage of women who perceive conflict is given by those who have children aged 18 and live at home (23.3%), 0–6 years (22.2%) and 7–11 years (20%). Percentages for the other age groups are 12.2% of those who have children between 15 and 18 years and 11.11% of those who have children between 12–14 and 18 years and do not live with them.

The main causes of family conflicts are related to: cooking and cleaning the house (33.9%), lack of privacy (25.2%), previous family problems worsened by imprisonment at home (22.5%), absence of division between working and non-working time (21.7%), use of on-line communication devices (16.1%), observance of the rules (15.7%), relationship with adult children -due to study, time spent on on-line games, use of social networks etc. (13.1%), children's homework (11.1%), childcare management (9.8%), economic problems (9.1%) and sexuality (7.7%).

Furthermore, the Chi-square analysis revealed a significant relationship with some of the different causes of conflict and the age of the children, as shown in Table 1.

**Table 1:** Causes of conflict and age of children.

**Causes of conflict**	**Age of children**	**%**	**df**	**χ^**2**^**	***P***	**Cramér's V**
Childcare management	0–6 years	42.9	5	34.72	0.000	0.55
	7–11 years	42.9				
	12–14 years	3.6				
	15–18 years	3.6				
	Over 18 years old and living at home	3.6				
	Over 18 years old and not living at home	3.6				
Children's homework	0–6 years	6.7	5	71.8	0.000	0.78
	7–11 years	56.7				
	12–14 years	26.7				
	15–18 years	10				
	Over 18 years old and living at home	0				
	Over 18 years old and not living at home	0				
Relationship with adult children	0–6 years	0	5	21.64	0.000	0.40
	7–11 years	11.5				
	12–14 years	15.4				
	15–18 years	23.1				
	Over 18 years old and living at home	42.3				
	Over 18 years old and not living at home	7.7				
Absence of division between working and non-working time	0–6 years	33.3	5	17.13	0.002	0.37
	7–11 years	25.9				
	12–14 years	0				
	15–18 years	22.2				
	Over 18 years old and living at home	14.8				
	Over 18 years old and not living at home	3.7				
Use of on-line communication devices	0–6 years	20	5	11.4	0.03	0.30
	7–11 years	30				
	12–14 years	20				
	15–18 years	10				
	Over 18 years old and living at home	20				
	Over 18 years old and not living at home	0				
Cooking and cleaning the house	0–6 years	26.7	5	11.39	0.04	0.30
	7–11 years	20				
	12–14 years	11.1				
	15–18 years	15.6				
	Over 18 years old and living at home	22.2				
	Over 18 years old and not living at home	4.4				

Moreover, the family conflict manifested itself with frequent quarrels (29.8%), isolation meant as keeping a muzzle, not speaking, withdrawing from the relationship, etc. (20.4%), with verbal violence (7.6%), psychological violence (2.3%), relationship control (1.8%), other (9.7%), while for the remaining cases there was no conflict.

As for the perception of family conflict, it emerges that the major conflict is with the partner; moreover the *t*-test for paired samples shows how the perception of the conflict has changed during the lock-down period compared to the previous period in relation to the parental figures and to the brothers/sisters, with whom it seems to have significantly decreased, this is obviously given by the fact that during the lock-down the moments of meeting and possible conflicts with non-resident family members were considerably reduced; while with the partners and the children appears to have remained unchanged (Table 2).

**Table 2:** Perception of conflict before and during the lock-down with the people women live with.

		***N***	**M(SD)**	***t***	***p***	**Cohen's *d***
Partner	Before	245	2.02 (1.03)	0.000	1.00	
	During		2.03 (1.18)			
Children	Before	173	1.82 (1.03)	0.33	0.74	
	During		1.80 (1.00)			
Parents	Before	217	2.01 (1.05)	3.94	0.000	0.27
	During		1.80 (1.06)			
Brothers/sisters	Before	201	1.83 (1.00)	4.51	0.000	0.32
	During		1.60 (0.93)			

### Neighborhood Relations and Collective Solidarity Initiatives

The lock-down also had effects on the dimension of the neighborhood: 52.2% of women said they talk to neighbors from the balcony, 46.7% said they no longer frequent their neighbors in their homes, 15.9% highlighted the exchange of information, 9.7% say they shop for each other while 9.2% exchange home-cooked food products, another 9% say they have come into contact with neighbors they did not know before, alongside these positive dimensions there are also two types of rather negative relationships: mutual control to ensure that the quarantine rules are respected (4.8%) and an increase in conflict (2.7%). Regarding the collective solidarity initiatives, most women report having joined in with solidarity initiatives (72.5%), most of them claim to have participated in one activity (30.1%), two (26.5%), three (11.1%), or four (4.9). In 36.3% of cases, they “shared literary/musicalF/cinematographic advice,” in 33.3% they “did the shopping for someone belonging to the categories most at risk,” in 26.1% “singing while looking out on the balcony,” in 19.6% “buying medicines for someone belonging to the categories most at risk,” in 17.3% they “created on-line content to entertain those who were at home.” Furthermore, most of the women declare that they were part, during the first lock-down in Italy, of one or more communities that came together on-line through new forms of rituals such as sport/music/dance/meditation/wellness (20.7%), discussions/workshops (18.2%), playing together (9.8%), celebrating anniversaries (6.0%), aperitifs/dinners (3.0%) or reading (2.4%).

## Discussion

The first Italian national lock-down started on 21 February 2020 and lasted until 3 May 2020, severely restricting citizens' freedom in order to safeguard public health.

In our sample, approximately one third of women declare an income lower than before and in half of the cases sustained from state aid, highlighting how economic suffering has also deeply affected the female gender (62–64).

During this period, the home and family relationships are perceived as a safe place, with the fear of being contaminated by the virus remaining outside the home, which accordingly becomes a protected place to take refuge. Clearly, this narrative excludes situations of domestic violence in which neither home nor forced cohabitation becomes a safe place and condition (26, 65). Outside the confines of the home, women implement recommendations and adopt behavior to prevent contagion by using personal health protection means, limiting or completely avoiding going out, washing hands frequently, shopping on-line, but also thinking about quality of life and physical well-being, for example by doing physical activity at home, including by connecting to on-line courses. Within this scenario, the theory of self-determination (66, 67) finds strength in a gender perspective that emphasizes how individuals are proactive or passive depending on the social conditions in which they are involved and could become a good form of interpretation and support for decision makers. It is a well-known fact that the theory of self-determination emphasizes how the type or quality of a person's motivation to follow recommendations and implement recommended behavior is more important than the amount of motivation to predict significant results also in relation to psychological health and well-being (68). In particular, we see how the autonomous motivations in which people identify themselves, such as the value of distancing and use of protective devices, and which they would ideally integrate in their sense of self, compared to those controlled and imposed, produce greater adhesion and therefore develop better psychological health. Indeed, the messages proposed by the government and the WHO tried to act on this motivational level, trying to involve citizens in the choices, internalizing values and sense of individual responsibility according to an active citizenship taking the perspective of social and intergenerational solidarity, shifting attention away from self-protection and toward the protection of the community as a whole (16) and that, from the data collected, women seem to have grasped.

However, precisely because the home is considered a safe environment, physical distancing is not implemented there, even though a fair percentage of women (around 15%) reveal that they avoid closer contacts such as kisses and hugs with people living together. The data collected through the IOS scale, which indicated the degree of closeness to each other, also showed that the sense of closeness and of “us” was higher when women indicated a person they considered close than a friend, like a neighbor, highlighting a sense of increasing social distancing from intimate to social relationships. This data is even more worrying if we compare it with the data collected before the pandemic where, while the sense of closeness with the person considered as close has not changed, for all other situations (friend, neighbor, etc.) the scores obtained are, respectively, lower showing a greater sense of distance with all those outside an intimate relationship. It should be emphasized that the sense of closeness and of “us” was not to be understood in a physical sense but rather in a psychological and emotional one: the lock-down period would therefore seem to have also affected the relational dimension as a loss of recognition of both friendly and neighborhood ties and relationships, in the face of the fact that women were in any case promoters or participants in a good percentage of solidarity and collective actions, as emerges from the above data. The role of women, in literature, has already been classified as one of promoters of psychological well-being and healthy communities, acting as creators of relational well-being within their life contexts (33).

In addition, our data shows a strong resilience of family relationships highlighting how women, in most cases, considered it important to face the lock-down with their family members or in a couple with their partner, especially in those situations where the previous relational dynamics were perceived as positive and satisfactory. It is clear from the reasons given by the women that the family was seen as an aid against the loneliness of lock-down and, where relationships were already positive, also an opportunity to spend time together outside the frenzy of everyday life. Forced isolation therefore came as an opportunity to rediscover family ties and to “do” something together. The moments of greater sharing in the family reveal a very articulated daily routine that combines routine situations such as eating meals or watching TV programmes with more creative activities such as cooking together, playing board games, playing sports, gardening, musical activities (singing, dancing) to name but a few that filled the days spent at home during the lock-down.

However, in the face of a perception of family relationships, despite the complex situation of using the rooms of the house in a new way, a high percentage of women (73.4%) claim that they experienced a dimension of family conflict. This situation appears to be related to the age of the children especially for those who had adult children still living at home or children in the 0–6 and 7–11 age groups. In these cases it is above all the management of daily life and the specific needs of non-autonomous children that has put the female gender, which is more involved in these activities than fathers, to the test (49, 69); in particular, needs in respect of material care, entertainment and also the management of distance learning have emerged. The significant relationship between family conflict perception and children's age also confirms earlier research carried out by us in the same period on a different sample and with a different data representation tool (49). As is well-known in literature, adolescence is a phase of the life cycle that is difficult to manage in general, but clearly all this has been amplified by the forced cohabitation between parents and children who normally spend a lot of time away from home in total autonomy and together with their peer group. While some authors (62, 70, 71) indicate that in part the lock-down was also an opportunity to rediscover relations between the different generations, it is plausible that some tensions have increased.

The main causes of family conflicts were related to routine domestic activities, preparing meals, cleaning the house, tidying up etc. These activities, although on the one hand presenting an opportunity to share and to spend time together, on the other hand, could be perceived as duties to be fulfilled. Activities that in pre-pandemic situations, many women and families handled turning to external services in order to reduce the burden of domestic and care tasks in the management of everyday life, especially in cases where both partners worked. Another significant aspect perceived by women as a source of conflict was the lack of privacy; the forced and continuous sharing of domestic space (not always adequately large enough to guarantee all family members a place of their own) which in many cases has also become the workplace has created tensions and conflicts between all living together.

These aspects also emerged in research that showed how COVID-19 changed the daily routine of families (72), who found themselves forced to share a restricted space for a long period of time, carrying out activities within it, for which another location was previously destined. Therefore, the boundaries between home and work blurred, and families are experiencing a particularly stressful.

However, our data shows that conflict situations have not resulted in forms of extreme conflict but instead have been mainly concentrated in an increase of quarrels or withdrawal behavior from the relationship with the other, such as sulking or not talking.

It is also highlighted that the most commonly experienced conflict is the one with the partner or children, which in any case remains unchanged compared to the period before the lock-down, underlining a resilience of family relationships.

Shifting the focus of reading the data outside of women's family relationships shows that the lock-down also impacted the size of the neighborhood in ways some of which we have already highlighted with the results on the proximity IOS scale; although 52.2% of women say that they talk to their neighbors from the balcony, in line with the scores on the IOS scale, 46.7% state that they no longer frequent their neighbors in their homes, underlining that forced distancing which was indeed regulated by legislation but also by fears of contagion that was well-represented by the choice to represent these links as distant circles through the IOS scale.

It is evident from the results that communities, and neighborhoods themselves, have undergone and are continuing to undergo significant changes due to COVID-19 (82). At the same time, however, as also emerges from Glover's study (73), a social connection as a neighborhood has been rediscovered albeit with the necessary safety distance.

As regards collective solidarity initiatives, most women report to have joined in solidarity initiatives (72.5%), showing despite the distancing, a perceived desire to maintain ties and play an active part in community life. Many women also participate in one or more on-line communities in order to create family-based sharing and use the time available in new social and relational ways.

However, this data seemed to show a level of home environmental safety in which everything outside lost the contours of normality while everything inside assumed safe boundaries where one could protect oneself, yet today with the second wave of the epidemic the virus has entered our homes, families relationships are the highest risk. The danger of making the weakest people sick by meeting with them again returns and the issue of social responsibility and health citizenship re-emerges.

It would appear to us that these recommendations once again reinforce the idea of the family living together as a group of trust. Given voice to their point of view as women, mothers and workers facing an unprecedented experiential crisis, it allowed us to outline an interesting exploratory framework while aware of the limits of using the methodology used. In fact, the use of both random cascade sampling and online questionnaires may have hindered a wider and more diversified participation among the population; however, due to social distancing, the online data collection strategy was considered the only feasible one, which made it possible to quickly reach a rather large and geographically distributed population on the Italian territory.

## Conclusion

The point of view of women, during the first lock-down for COVID-19 in Italy, highlights interesting reflections to be submitted to the scientific debate on the issues in question and to the attention of political debate and decision makers. The article examines the matter from a gender perspective that takes into account the point of view of women some of the problems that the Italian community had to face in the period of forced and prolonged cohabitation during the lock-down. The initial questions concern the central topic of social distancing, the maintenance of family relations, the dimension of family conflict and neighborhood relations in the dimension of collective solidarity. In the text we have discussed in detail the results obtained in every aspect of the research, and here we would like to just briefly focus attention on the multidisciplinary approach that has been the hallmark of this research and which is a strong point in terms of the effectiveness of reading the data that has emerged and which makes it possible to better identify critical points that can be a starting point for hypothesizing interventions aimed at improving political choices and intervention in a situation that continues to last indefinitely and unpredictably. The spread of the virus is showing signs of renewed vigor throughout Europe and Italy is no exception; this leads many virologists to talk about a second wave of pandemic that sees in the fragility and critical issues that emerged in our work the basis on which to intervene to avoid the increase of closure in the family dimension that now seems less secure than then and in which the fragility of the bonds inside and outside the home seem to become aspects on which to open important reflections so as not to lead women to a concrete and not very constructive, isolation. Family, social and neighborhood ties return to the center of the fear of contagion and become the protagonists of the strategies to be put in place to face the pandemic once again, which seems to have regained strength precisely in relation to the relaxation of relational precautions and the consequent abandonment of the “collective pact” that assumes health as a public asset. It would appear to us that we can interpret this as an awareness of being part of what Beck and Gernsheim (74) call the existential community of global destiny.

We believe that the results of our investigation may constitute important points of reflection for decision-makers and politicians in planning interventions and in producing compliance messages of action in which the idea of citizenship does not conflict with health and economic rights.

## Data Availability Statement

The raw data supporting the conclusions of this article will be made available by the authors, without undue reservation.

## Ethics Statement

The studies involving human participants were reviewed and approved by University of Genova. The participants provided their written informed consent to participate in this study.

## Author Contributions

RP, NR, IC, and FL conceived the original idea of the study and supervised the findings of this work. NR and IC contributed to data processing and analysis. All authors wrote and organized the manuscript, in particular RP and FL developed the introduction and the first and second paragraph, and NR wrote the third paragraph. While NR and IC presented the methology, procedure, and data section. All authors discussed the results, reviewed the document, and approved the final version for submission.

## Conflict of Interest

The authors declare that the research was conducted in the absence of any commercial or financial relationships that could be construed as a potential conflict of interest.

## References

[1] Porter D. Health Citizenship. Essays in Social Medicine and Biomedical Politics. San Francisco, CA: University Of California Medical Humanities Press (2011).

[2] Baldassar L Merla L. Transnational Families, Migration and the Circulation of Care. Understanding Mobility and Absence in Family Life. London: New York Routledge (2014).

[3] Lagomarsino F Pagnotta C. Sull'alterità dei giovani latinoamericani. Sessualità adolescente a Genova [About Latin American young otherness. Adolescent sexuality in Genoa]. In: Ambrosini M, Torre AT, editors. Settimo Rapporto sull'immigrazione a Genova [Seventh Report on Migration in Genoa]. Genova: Il Melangolo. (2010). p. 119–152.

[4] Pedone C. Rupturas y continuidades de los roles de género en contextos migratorios transnacionales. Relatos sobre sexualidad y salud reproductiva de los hijos e hijas de la inmigración ecuatoriana en Cataluña. Papeles CEIC. (2014) 2:1–38. 10.1387/pceic.12968

[5] Beck U. La Società del Rischio. Verso una Seconda Modernità. Roma: Carocci (1986).

[6] De Singly F. Le soi, le Couple et la famille. Paris: Nathan (1996).

[7] De Singly F. Les uns avec les autres. Quand l'individualisme crée du lien. Paris: Armand Colin (2003).

[8] Sennett R. Il declino dell'uomo Pubblico. Milano: Mondadori (1976).

[9] Magaraggia S. Il genere nelle famiglie. In: Satta C, Magaraggia S, Camozzi I, editors. Sociologia Della Vita Famigliare. Soggetti, Contesti e Nuove Prospettive. Roma: Carocci (2020). p. 55–88.

[10] Ammar A Brach M Trabelsi K Chtourou H Boukhris O Masmoudi L. Effects of COVID-19 home confinement on eating behaviour and physical activity: results of the ECLB-COVID19 international online survey. Nutrients. (2020) 12:1–13. 10.3390/nu12061583 32481594 PMC7352706

[11] Ammar A Chtourou H Boukhris O Trabelsi K Masmoudi L Brach M. COVID-19 home confinement negatively impacts social participation life satisfaction: a worldwide multicenter study. Int J Environ Res Public Health. (2020) 17:1–17. 10.3390/ijerph17176237 32867287 PMC7503681

[12] Quaranta I. Prospettiva Globale e Partecipazione Comunitaria. Atlante Treccani. (2020). Available online at: https://www.treccani.it/magazine/atlante/cultura/Storie_Virali_Prospettiva_globale.html (accessed September 5, 2020).

[13] Paparella N Limone P Cinnella G. Pandemia. Apprendere per prevenire. Bari: Progedit (2020).

[14] Beneduce R. Storie Virali. Le lezioni di una pandemia. Atlante Treccani. (2020). Available online at: http://www.treccani.it/magazine/atlante/cultura/Le_lezioni_di_una_pandemia.html. (accessed April 5, 2020).

[15] Galasso V Vincent Pons V Profeta P Becher M Brouard S Foucault M. Gender differences in COVID-19 attitudes and behavior: panel evidence from eight countries. Proc Natl Acad Sci USA. (2020) 117:27285–91. 10.1073/pnas.2012520117 33060298 PMC7959517

[16] Cheng KK Lam TH Leung CC. Wearing face masks in the community during the COVID-19 pandemic: altruism and solidarity. Lancet. (2020). 10.1016/S0140-6736(20)30918-1. Available online at: https://www.thelancet.com/journals/lancet/article/PIIS0140-6736(20)309181/fulltext#articleInformation PMC7162638 32305074

[17] Moretti C. Storie Virali. Responsabilità e Colpevolezza. Atlante Treccani. (2020) Available online at: https://www.treccani.it/magazine/atlante/cultura/Storie_Virali_Responsabilita_e_colpevolezza.html (accessed May 20, 2020).

[18] Nikolas RN. Governing the Soul. The Shaping of the Private Self. London: Free Associate Books (1989).

[19] Saitta P. Storie Virali. Tempi di eccezione? Atlante Treccani. (2020) Avaialble online at: https://www.treccani.it/magazine/atlante/cultura/Storie_virali_Tempi_di_eccezione.html (accessed April 15, 2020).

[20] Guigoni A. #iocucinoacasa. Lockdown italiano: pratiche culinarie in quarantena. In: Guigoni A, Ferri, R, editors. Pandemia 2020. La vita Quotidiana in Italia con il Covid-19. M&J Publishing House (2020). p. 143–50. Available online at: http://www.fondazionestudistoriciturati.it/sorget/pubblicazioni/

[21] Cicchetti D. Annual research review: resilient functioning in maltreated children– past, present, and future perspectives. J Child Psychol Psychiatry. (2013) 54:402–22. 10.1111/j.1469-7610.2012.02608.x 22928717 PMC3514621

[22] Masten AS Monn AR. Child and family resilience: A call for integrated science, practice, and professional training. Family Relations. (2015) 64:5–21. 10.1111/fare.12103

[23] Henry CS Sheffield Morris A Harrist AW. Family resilience: moving into the third wave. Fam Relat. (2015) 64:22–43. 10.1111/fare.12106

[24] Grevenstein D Bluemke M Schweitzer J Aguilar-Raab C. Better family relationships—higher well-being: the connection between relationship quality and health related resources. Mental Health Prevention. (2019) 14:1–8. 10.1016/j.mph.2019.200160

[25] Scrimin S Osler G Pozzoli T Moscardino U. Early adversities, family support, and child well-being: the moderating role of environmental sensitivity. Child Care Health Dev. (2018) 44:885–91. 10.1111/cch.12596 30051489

[26] Moffa G Chirivì M. La violenza di genere confinata tra le pareti domestiche du-rante il lockdown. Cult Studi Soc. (2020) 5:559–67.

[27] Istat. Violenza di Genere al Tempo del Covid-19: le Chiamate al Numero Verde 1522. Roma: Istat 13 (2020).

[28] Migliorati L. Un Sociologo Nella Zona Rossa. Rischio, Paura, Morte e Creatività. Ai Tempi di covid-19. Milano: Franco Angeli. (2020).

[29] Magatti M. Libertà Immaginaria. Le Illusioni del Capitalismo Tecno-nichilista. Milano: Feltrinelli (2009).

[30] Bauman Z. Voglia di Comunità. Bari: Laterza (2001).

[31] Anderson B. Comunità Immaginate. Roma: Manifestolibri (1996).

[32] Appadurai A. Modernity at Large. Cultural Dimention of Globalitation. Minneapolis-London: University of Minnesota Press (1996).

[33] Rania N Migliorini L Zunino A Lena C. Psychological well-being and healthy communities: Women as makers of relational well-being by social street strategies. J Prev Interv Community. (2020) 48:161–73. 10.1080/10852352.2019.1624355 31190636

[34] Palumbo B. Storie Virali. Ibridi, Atlante Treccani. (2020). Available online at: http://www.treccani.it/magazine/atlante/cultura/Storie_virali_Ibridi.html?fbclid=IwAR3aoSvxtct6JBDYc7-FyJBMyB3q8Xw4uJKuPjd-b69JxZ7701BbLNbK-FA (accessed April 20, 2020).

[35] Smith ML Steinman LE Casey EA. Combatting social isolation among older adults in a time of physical distancing: the COVID-19 social connectivity paradox front. Front Public Health. (2020) 8:403. 10.3389/fpubh.2020.00403 32850605 PMC7396644

[36] Porter D. Health Citizenship. Essays in Social Medicine and Biomedical Politics. San Francisco, CA: University Of California Medical Humanities Press (2011).

[37] Schirripa G. Storie Virali. Colera e incubi (con uno sguardo a oggi). Atlante Treccani. (2020) Available online at: http://www.treccani.it/magazine/atlante/cultura/Storie_virali_Colera_e_incubi_con_uno_sguardo_a_oggi.html (accessed June 15, 2020).

[38] Isin EF. Citizenship in flux: the figure of the activist citizen. Subjectivity. (2009) 29:367–88. 10.1057/sub.2009.25

[39] Bourdieu P. Il Dominio Maschile. Milano: Feltrinelli (1998).

[40] Busoni M. Genere Sesso, Cultura. Uno Sguardo Antropologico. Roma: Carocci (2000).

[41] Abbatecola E Stagi L. Pink Is the New Black. Stereotipidi Genere Nella Scuola dell'infanzia. Torino: Rosenberg & Sellier (2017).

[42] Migliorini L Rania N. Il genere come contesto: verso una psicologia di genere tout court, In: De Piccoli N, Rollero C, editors. Sui generi: identità e stereotipi in evoluzione? CIRSDe, Centro Interdisciplinare di Ricerche e Studi delle Donne e di Genere, Università degli Studi di Torino. Torino (2018). p. 175–84.

[43] Bond M Serrano-Garcia I Keys C. Handbook of Community Psychology, Volume 1: Theoretical Foundations, Core Concepts, and Emerging Challenges. Washington DC: American Psychological Association Press (2017). p. 1201.

[44] Rollero C Gattino S De Piccoli N. A gender lens on quality of life: the role of sense of community, perceived social support, self-reported health and income. Soc Indicat Res. (2014) 116:887–98. 10.1007/s11205-013-0316-9

[45] Yuval-Davis N. Intersectionality and feminist politics. Euro J Women Studies. (2006) 13:193–209. 10.1177/1350506806065752

[46] Lewin K. Teoria e Sperimentazione in Psicologia Sociale. Bologna: il Mulino (1972).

[47] Levine M Perkins D Perkins D. Principles of Community Psychology. Perspective and Application. Oxford: University Press (2005).

[48] Ausín B González-Sanguino C Castellanos MÁ Muñoz M. Gender-related differences in the psychological impact of confinement as a consequence of COVID-19 in Spain. J Gender Stud. (2020) 30:29–38. 10.1080/09589236.2020.1799768

[49] Rania N Coppola I Lagomarsino F Parisi R. Lockdown e ruoli di genere: differenze e conflitti ai tempi del Covid-19 in ambito domestico. La Camera Blu. Rivista studi di genere, (2020) 22:35–60. 10.6092/1827-9198/6813

[50] Migliorini L Rania N De Piccoli N. Gender matters and the challenge for improving community health and well-being. J Prev Interv Community. (2020) 48:113–20. 10.1080/10852352.2019.1624351 31184545

[51] Rania N Migliorini L Zunino A Bianchetti P Vidili MG Cavanna D. La riabilitazione oncologica: qualità della cura e benessere psicologico del paziente. Salute Soc. (2015) 2:60–73. 10.3280/SES2015-002005

[52] Rania N Migliorini L Vidili MG Bianchetti P Forno G Cavanna D. Exploring well-being and satisfaction with physiotherapy efficacy: an Italian study of cancer patients. Mediter J Clin Psychol. (2018) 6:1–21. 10.6092/2282-1619/2018.6.1841

[53] Rania N Migliorini L. Vivere con la mutazione genetica BRCA: implicazioni psicosociali e percezione del rischio di cancro. Salute Soc. (2015) 2:100–13. 10.3280/SES2015-002008

[54] Battistuzzi L Franiuk M Kasparian N Rania N Migliorini L Varesco L. A qualitative study on decision-making about BRCA1/2 testing in Italian women. Eur J Cancer Care. (2019) 28:1–9. 10.1111/ecc.13083 31056822

[55] Regmi PR Waithaka E Paudyal A Simkhada P van Teijlingen E. Guide to the design and application of online questionnaire surveys. Nepal J Epidemiol. (2016) 6:640–4. 10.3126/nje.v6i4.17258 28804676 PMC5506389

[56] Aron A Aron EN Smollan D. Inclusion of other in the self scale and the structure of interpersonal closeness. J Personal Soc Psychol. (1992) 63:596–612. 10.1037/0022-3514.63.4.596

[57] Gächter S Starmer C Tufano F. Measuring the closeness of relationships: a comprehensive evaluation of the 'inclusion of the other in the self' scale. PLoS ONE. (2015) 10:e129478. 10.1371/journal.pone.0129478 26068873 PMC4466912

[58] Wang C Pan R Wan X Tan Y Xu L Ho CS. Immediate psychological responses and associated factors during the initial stage of the 2019 coronavirus disease (COVID-19) epidemic among the general population in China. Int J Environ Res Public Health. (2020) 17:1–25. 10.3390/ijerph17051729 32155789 PMC7084952

[59] Rodríguez-Rey R Garrido-Hernansaiz H Collado S. Psychological impact and associated factors during the initial stage of the coronavirus (COVID-19) pandemic among the general population in Spain. Front Psychol. (2020) 11:e1540. 10.3389/fpsyg.2020.01540 32655463 PMC7325630

[60] Leech NL Onwuegbuzie AJ. Beyond constant comparison qualitative data analysis: Using NVivo. School Psychol Q. (2011) 26:70–84. 10.1037/a0022711

[61] Glaser BG Strauss AL. The Discovery of Grounded Theory: Strategies for Qualitative Research. Roma: Armando (2009).

[62] Centro di Ateneo Studi e Ricerche sulla Famiglia Università Cattolica del Sacro Cuore. La famiglia sospesa. Milano: Vita e Pensiero (2020).

[63] Save the Children. Riscriviamo il Futuro. L'impatto del Coronavirus Sulla Povertà Educativa. Roma: Save the Children (2020).

[64] Ferrario T Profeta P. Covid: Un Paese in Bilico tra Rischi e Opportunità Donne in Prima Linea. Laboratorio Futuro, Istituto Toniolo. (2020). Available online at http://laboratoriofuturo.it/ricerche/covid-un-paese-in-bilico-tra-rischi-e-opportunita-donne-in-prima-linea/ (accessed February 19, 2021).

[65] Albanesi C. Prima le donne e i bambini? La Camera Blu. (2020) 22:118–27. 10.6092/1827-9198/7089

[66] Ryan RM Deci EL. Self-determination theory and the facilitation of intrinsic motivation, social development, and well-being. Am. Psychol. (2000) 55:68–78. 10.1037/0003-066x.55.1.68 11392867

[67] Migliorini L Cardinali P Rania N. How could self-determination theory be useful for facing health innovation challenges? Front.Psychol. (2019) 10:1870. 10.3389/fpsyg.2019.01870 31474910 PMC6702320

[68] Deci EL Ryan M. Self-determination theory: a macrotheory of human motivation, development, and health. Can Psychol. (2008) 49:182–5. 10.1037/a0012801

[69] Saban Orsini M Barone C. *100 d*í*as Covid*. Tareas de Cuidado y Productividad. Buenos Aires: CESBA (2020).

[70] Forum Associazioni Familiari. Le Famiglie e L'emergenza covid-19 una Fotografia Attuale, RCS Sfera Mediagroup e Forum Delle Associazioni Familiari. (2020). Available online at https://www.forumfamigliepuglia.org/wp-content/uploads/2020/07/Indagine-Famiglie-report.pdf (accessed February 16, 2021).

[71] Behar-Zusman V Chavez JV Gattamorta K. Developing a measure of the impact of COVID-19 social distancing on household conflict and cohesion. Fam Proc. (2020) 59:1045–59. 10.1111/famp.12579 32621755 PMC7362045

[72] Russell BS Hutchison M Tambling R Tomkunas AJ Horton AL. Initial challenges of caregiving during COVID-19: caregiver burden, mental health, and the parent-child relationship. Child Psychiatry Human Dev. (2020) 51:671–82. 10.1007/s10578-020-01037-x 32749568 PMC7398861

[73] Glover TD. Neighboring in the time of coronavirus? Paying civil attention while walking the neighborhood. Leisure Sci. (2020). 10.1080/01490400.2020.1774014. Available online at: https://www.tandfonline.com/doi/full/10.1080/01490400.2020.1774014?scroll=top&needAccess=true

[74] Beck U Beck-Gernsheim E. L'amore a Distanza. Il Caos Globale Degli Affetti. Bari: Editori Laterza (2012).

